# 'Stability' of Assessment: Extending the Utility Equation

**DOI:** 10.15694/mep.2021.000155.1

**Published:** 2021-06-04

**Authors:** Tarun Sen Gupta, Eugene Wong, Deepak Doshi, Richard Hays

**Affiliations:** 1 James Cook University and The Australian College of Rural & Remote Medicine; 2 The Australian College of Rural & Remote Medicine; 3 James Cook University

**Keywords:** assessment, utility equation, stability

## Abstract

This article was migrated. The article was marked as recommended.

The COVID-19 pandemic has brought many challenges to medical education, necessitating a rapid transition to digital delivery. The widespread move to online exams has introduced novel risks, including the risk of catastrophic IT failure. These are not ‘black swan’ events - something so unexpected and devastating that we could not anticipate them and prepare accordingly. The phrase ‘black elephants’, a cross between a black swan and ‘the elephant in the room’ has been coined to describe these events.

Moving to high-stakes online examinations introduces another element that needs to be considered and managed: the ‘stability’ of the assessment format used. This dimension incorporates notions of ‘platform reliability’ and ‘internal risk management’ and can be caused by both unplanned events eg IT failures, and planned events like security breaches

Developing approaches to mitigate this new risk suggests another dimension to the well-known assessment ‘utility equation’: stability of the platform used. This paper explores the concept of stability from the perspectives of educational institutions and candidates and offers some approaches to achieving stability. The delivery of assessment in the digital age, requires the ‘utility equation’ to be recalibrated and establishment of a new “sweet spot” for each assessment program.

## Introduction

COVID-19 has affected every aspect of life globally since 2020. Medical education has not been immune. Responses to the threat posed by COVID have included withdrawal of students from clinical and educational settings, modification of activities, and substantial changes to education design and assessment. These have involved considerable ingenuity and innovation, and led to a burgeoning literature ( Hays *et al.,* 2020).

Many educational institutions moved their activities online, which has posed significant challenges but also provided some opportunities. Due to pressured timelines many programs simply delivered existing curriculum content using available platforms, which are more repositories of information rather than facilitators of interactive learning. Online technology use in assessment was generally limited to specific assessment methods (e.g. multiple choice questions) and with limited numbers. Instead of technology being ahead of practice, current hardware and software was challenged to support the transition to almost entirely on-line learning and assessment. The continued expansion of information technology (IT) means e-platforms are constantly improving and becoming more efficient, with the pandemic stimulating a flurry of technological innovations and advances ( Perlroth, 2021). Yet the global nature of the pandemic put strain on existing information communication infrastructure and capacity, as networks were being utilised 24/7 by virtually everyone worldwide. Internet traffic was estimated to have increased by 30-40% compared to pre-pandemic levels ( Perlroth, 2021). Users of platforms such as Zoom and Microsoft Teams more than tripled in the first few months of the pandemic ( Kastrenakes, 2020; Warren, 2020).

Significant issues also arose in accessing online systems away from usual workplaces. Internet access, bandwidth and speed and the specifications of individual computer equipment off campus varied widely.

In assessment, where security, timing and efficiency in managing large amounts of data are paramount, one major risk to manage is the risk of catastrophic IT failure, of which there were several examples during 2020. These are not ‘black swan’ events - something so unexpected and devastating that we could not anticipate them and prepare. The business literature points out that while it is tempting to regard the pandemic as a black swan event, we have long appreciated the risk of pandemics, and boards can (and should) ‘plan for the disasters that no one wants to think about.’ ( Burchman and Jones, 2020)

This has led to the coining of the phrase ‘black elephants’, a cross between a black swan and ‘the elephant in the room’ ( Burchman and Jones, 2020). The 2008 financial crisis was one such black elephant. We are likely to see further catastrophes - and crimes, perhaps related to cyber-attacks, IT failures and climate change due to global warming. Given the interconnectedness of global markets such events are likely to have far-reaching consequences beyond the local impacts.

In the business world, management leaders have a responsibility to ensure their business model is resilient, robust, and can survive threats posed by black elephants. Educational institutions too, have a responsibility to recognise and manage such threats. Other sectors such as banking, airlines and the military have similar high-stakes contexts and have been using the e-environment for decades. Any new venture will have attendant risks and potential disasters which demand mitigation strategies and a continuous improvement approach ( Burchman and Jones, 2020).

## Implications for assessment

In this paper the authors argue that moving to high-stakes online examinations introduces another element that needs to be considered and managed: the ‘stability’ of the assessment format used. This dimension could also be regarded as ‘platform reliability’ or ‘internal risk management’. In essence there is an additional risk that needs to be articulated, understood and managed in planning the different approach to delivering online examinations compared to face-to-face. For example, a traditional multiple choice examination delivered in person as a ‘pen and paper test’ has minimal but appreciable risks inherent in the format. Venue problems, power failure, severe weather events, or non-attendance of key participants are all events that can be anticipated and managed. In contrast, catastrophic IT events such as widespread disruption to connectivity, software failure or login problems may be less amenable to timely resolution on the day. While such events may be anticipated, the specific details are not predictable and thus less amenable to resolution in real-time.

Stability can be defined as ‘the quality or state of being steady and not changing or being upset in any way (= the quality of being stable)’ ( Oxford Learner’s Dictionaries, 2021). ‘Changes’ or ‘upsets’ can be caused by both unplanned events eg IT failures, and planned events like security breaches and other illicit activities. Even ‘sub-catastrophic’ events like a choppy video or audio dropouts can affect stability and compromise the assessment.

Van der Vleuten argues that assessment is more an instructional design issue than a measurement one, with potential problems related to educational, implementation or resource constraints. His utility model proposes conceptualisation of context-specific compromises, wherein various assessment characteristics are weighted differently according to the purpose and context of the assessment ( van der Vleuten, 1996).

The utility ‘equation’, intended as a conceptual model rather than an actuarial algorithm, can be written thus:

Utility of Assessment = Validity ^w1^ x Reliability ^w2^ x Cost ^w3^ x Acceptability ^w4^ x Educational impact ^w5^

Van der Vleuten acknowledges that many of these elements cannot be quantified, so it is not a new psychometric index, and there may be other criteria that could be included in the model such as transparency, meaningfulness, cognitive complexity, directness and fairness. Nevertheless, the model is helpful in highlighting the need for compromise decisions across these dimensions, according to context and local policy imperatives. For example, a very large number of items that samples widely is well known to improve reliability, but brings with it increased costs and perhaps lower acceptability. In other settings educational impact may be the most important factor, and therefore may be a paramount concern in the design of the assessment, and so forth.

The 2018 Ottawa Consensus Framework for Good Assessment reiterates these principles, and also proposes the further dimensions of *Equivalence* and *Catalytic effect* ( Table 1). The authors note they place ‘particular emphasis on the educational and catalytic effect of assessment.’ Educational Effect means, ‘The assessment motivates those who take it to prepare in a fashion that has educational benefit’, while Catalytic effect is defined as, ‘The assessment provides results and feedback in a fashion that motivates all stakeholders to create, enhance, and support education; it drives future learning forward and improves overall program quality.’ They further point out the importance in summative assessment of high-quality test material, credible standard-setting processes, and secure administration, arguing, ‘elements such as validity-coherence, reproducibility-consistency, and equivalence are paramount.’ ( Norcini *et al.,* 2018) Interestingly, security is not otherwise discussed in this framework. As outlined below, ‘security’ has both a general meaning (‘the state of being free from danger or threat’) and a more specific one (‘the safety of a state or organization against criminal activity such as terrorism, theft, or espionage’) - both of which are relevant ( Lexico, 2021).

**Table 1:** Framework for Good Assessment: Single Assessments

1. Validity or Coherence.
2. Reproducibility, Reliability, or Consistency.
3. Equivalence.
4. Feasibility.
5. Educational Effect.
6. Catalytic effect.
7. Acceptability.

Adapted from Norcini *et al.,* (2018).

COVID-19 has seen many changes to assessment, with a movement towards online assessment and relatively few face-to-face assessments ( Hays *et al.,* 2020). The risk inherent in running high stakes assessments online has been well recognized, with a number of documented “technical glitches” causing examinations to be rescheduled or postponed ( Visontay, 2020).

We therefore propose that the ‘stability’ of the online assessment platform used is another dimension to be considered and managed. This gives us an updated Utility equation in the time of COVID, viz

Utility = Validity ^w1^ x Reliability ^w2^ x Cost ^w3^ x Acceptability ^w4^ x Educational impact ^w5^ x Stability ^w6^

If we accept that all assessment is a compromise then this modification introduces another factor to be considered and managed. Given the need to trade off the various components, ‘100% ‘stability’ will be impossible to achieve, so we will have to consider ‘how much is enough?’

Stability embraces the notions of reproducibility/reliability/consistency, feasibility, and acceptability as noted in the 2018 Ottawa framework. It also encompasses the idea of adaptability, as risk management requires mechanisms for recovering rapidly or switching to other delivery methods.

‘Secure administration’ assumes a much greater importance in a peri-COVID world where online formats dominate, and security can mean much more than preventing unauthorised access or leakage.

## What does stability mean?

The ‘Stability’ of an online assessment approach relates to the reliability of the educational design and platform used (‘not changing or being upset’), and represents a form of internal risk management to ensure secure administration of the exam to all candidates. There are several interacting components:

• **Institutional.** These include staff training and expertise, communications, back-up plans, and risk management. Plans need to be in place to troubleshoot and manage contingencies. Failure to do this in the current environment constitutes a significant policy risk
• **Candidates.** Candidates typically have a diverse range of IT skills, IT infrastructure, and local IT support. A flexible and facilitative approach is needed to overcome potential user error, and user connectivity problems
• **Service Platform/IT solution**.The preferred IT solution needs to beuser-friendly, available across multiple devices, and able to accommodate unstable broadband access. Apart from infrastructure/connectivity risks there may be software glitches and counterparty risks of the service providers. This refers to the risk that the other party in a contract may not meet its obligations, eg service quality obligations, privacy obligations or even appropriate use of data. In the context of online assessment the educational institution depends on the reliability and trustworthiness of the online service provider/platform ( Clement, 2017).
• **Security.** The possibility of cyberattacks including denial of service, ransomware attacks and hacking must also be considered. Further risks relating to Integrity of information eg, unauthorized leaks and sharing of information digitally must also be considered as part of the security plan. In a rapidly evolving field we are seeing increasing sophisticated approaches to security; however as these approaches mature so, too, might the approaches of the cyber hackers and the unscrupulous.
• **Vendor after-sales support.** Given the high-stakes setting and complexity of the environment it may well be that real-time access to centralized support should form part of the after-sales support where a third-party vendor is used

**Figure 1:**
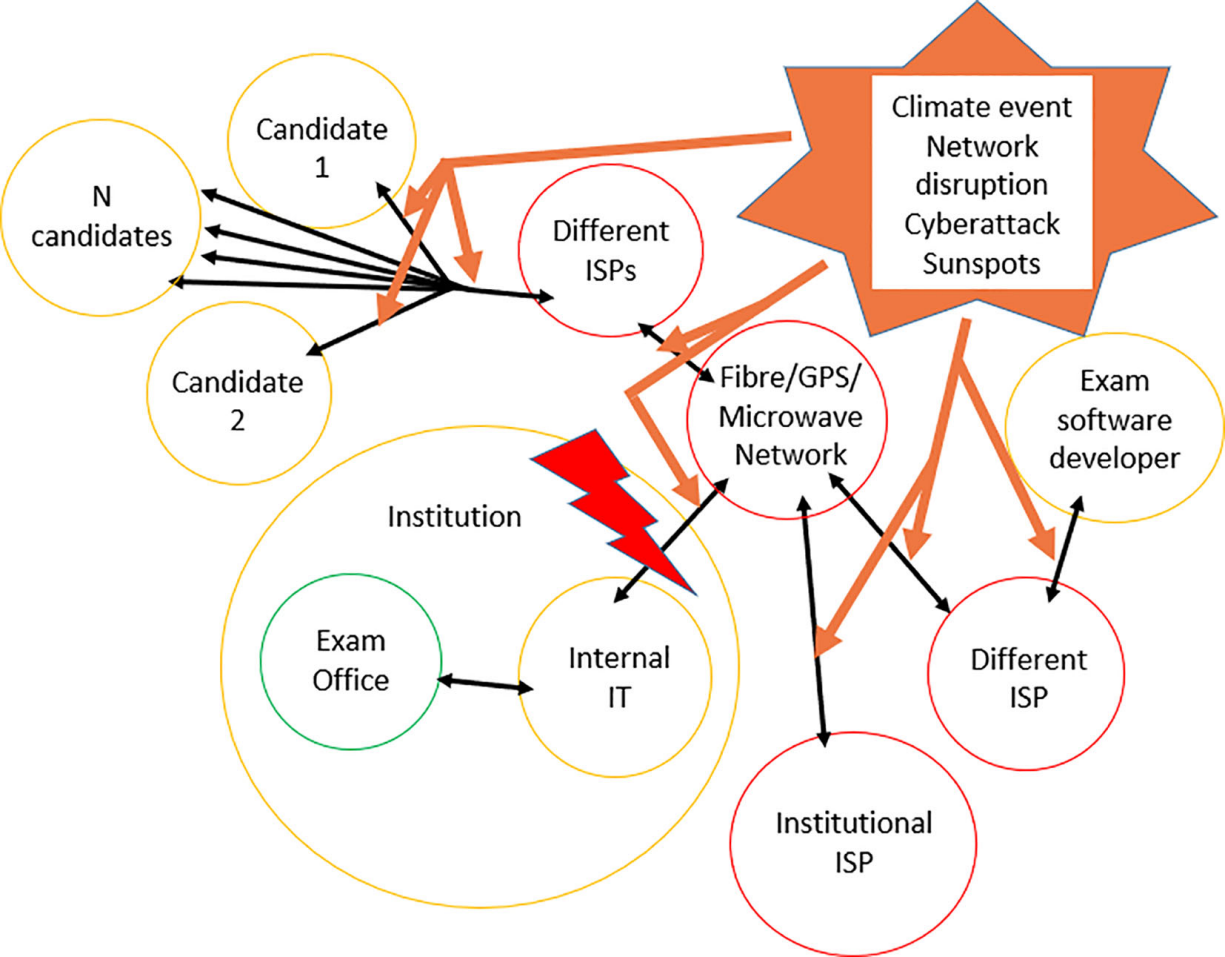
Risk managing stability in online assessment

Figure 1 illustrates the complexity of risk managing stability in online assessment. There may be multiple different Internet Service Providers (ISPs) and distribution networks. Faults with only one component may destabilise the entire assessment system. Many points of risk are partially or completely outside the control of the educational institution.

1. Green border = within control of educational institution; Yellow border = partial control only; Red border = no control

2. ISP = Internet Service Provider

Stability will be further enhanced by improving design to match assessment methods better to the available technology as well as guiding software developers to improve IT systems. In the time of COVID the platform of delivery is likely to be a decision-making factor in how assessments are designed and written, in contrast to the past.

Central to stable administration of an online examination is good communication between the many stakeholders. The educational institution needs to work closely with any third-party provider of the platform used and be in constant communication with candidates. While educators involved in assessment programs are likely to have some IT literacy, they may be not be technical experts. It is therefore challenging for the educational institution to anticipate and risk mitigate all the possible things that could go wrong, even if they have access to in-house IT expertise. Delivery of assessment should therefore be designed to match the constraints including the capabilities of technology and staff, rather than shoehorning old assessment modalities into new delivery platforms. Thus, there is an onus on institutions in the new digital age to robustly consider IT literacy and capacity as part of their risk management framework/systems at all levels of the organization from the Board down.

As discussed below, candidates do not just need to have the ability to access the platform in terms of bandwidth and hardware, IT skills and IT support; they need to be confident they can do so on the day.

## The importance of stability

There are many implications of stability - or the lack thereof - for educational institutions, trainees, training bodies and the community.

Failure of stability can lead to substantial reputation damage for the institution, not to mention the time and cost in putting things right, and potential legal risks. Staff, too, are at risk as they deal with a complex and changing scenario and have to communicate with distressed trainees.

Impact on candidates includes increased stress for them and their families, from the impact of the actual incident of examination instability, and from the psychological fatigue associated with the subsequent uncertainty and extended period of study due to delay in assessment. There are additional pecuniary and temporal implications (e.g. delay to qualification), and other psychological and professional consequences which may be difficult to measure (e.g. inability to obtain a desired post or career delay).

Training organizations and employing jurisdictions may be affected by delays in progression and graduation, which, in turn, have the potential to affect provision of services to the community. And if concerns around stability lead to adoption of a narrower range of assessment methods then this may have implications for competence at graduation and subsequent supervisory arrangements.

An Australian review identified a number of key concerns of junior doctors. These include redeployment, examination uncertainty, a bottle-necked workforce (i.e. possible reduction of new training places and job opportunities in 2021) and potential failure of career progression. The authors noted, ‘tensions between local workforce demands, experiences required for career progression, and the requirements of training, accreditation and registration bodies.’ ( Johnston *et al.,* 2020)

## How to achieve stability

Given the many factors involved in ensuring stability, there are also a variety of approaches needed. The approach, software and IT support need to be carefully chosen. Assessment methods need to be designed that make better use of all the functionality offered by the technology. For example, for current methods, could examinations be downloaded in advance to candidates’ computers in a secure encrypted compartment controlled by the institution, completed offline and transmitted for data management after completion? Could software that analyses qualitative text be adapted to automatically mark written text answers against model answers? Could simulation assessment be entirely remote and asynchronous? Could workplace-based assessment be conducted with candidates and patients only present and the candidate wearing a ‘headcam’, replacing OSCEs?

Candidates, too, need to be better prepared for all possibilities. Back-up plans that can be implemented rapidly need to be developed with appropriate command-and-control governance to make rapid decisions on the day. As with preparation for any major disaster, a well-structured disaster plan needs to be in place. Determine in advance what, if any, mitigation procedures may be required for some or all candidates.

One example to approaching this problem comes from the Australian College of Rural and Remote Medicine (ACRRM) which offers the world’s first and only Fellowship exam in Rural Medicine providing candidates the opportunity to participate in assessment in or close to their home location. The College uses multiple modalities in its assessment program, including multiple choice questions, multi-source feedback and case-based discussions ( Sen Gupta *et al.,* 2020). Work-based assessments such as multi-source feedback are readily delivered at a distance, while there are a number of established approaches to online delivery of multiple choice questions.

A major component of the ACRRM assessment program is the Structured Assessment using Multiple Patient Scenarios (StAMPS) examination, which blends the formats of an Objective Structured Clinical Examination (OSCE) and a traditional viva vocè examination. Since 2008 ACRRM has provided a videoconferencing option which allows candidates to remain in or near their home location, while the examiners meet a central location. Travel restrictions due to the COVID-19 pandemic meant that in 2020 both candidates AND examiners participated in StAMPS exclusively via videoconference.

A recent paper describes *Twelve Tips* for delivering high-stakes OSCE-style examinations via video tele-assessment, based on ACRRM’s extensive experience since 2008, both in tele-assessment in the rural context and with subsequent adaptations for the COVID environment ( Lewandowski *et al.,* 2020).

These tips noted the importance of preparation - of the cases, the examiners and the candidates. The authors described the importance of getting the IT platform right, based upon factors such as scale of the examination, cost, technical requirements, reliability, security features and IT support. They argued, ‘Above all it needs to be easy to use for examiners and candidates.’ And of course support staff, invigilators and many others are also involved.

The authors also observed the importance of vigilance with security, developing quality assurance processes and taking great care with logistics. They offered a number of recommendations on risk management, including a formal risk management strategy and concluded, ‘With current readily available technologies many assessments that traditionally were performed in person can now be performed via tele-assessment improving convenience, reducing travel time and costs, and allowing adaptation to necessities and impediments outside of the examination itself.’ ( Lewandowski *et al.,* 2020)

Nevertheless, exams can fail, both due to IT and non-IT factors. While the ACRRM StAMPS exam illustrates some lessons in how to achieve stability, ACRRM also experienced an examination failure in its September 2020 online MCQ exam. Only 36 of the total of 89 candidates were able to complete the exam due to an unexplained technical issue experienced on the day, despite a long history of administering online MCQ examinations and extensive prior testing and preparation. The College’s response included immediate and repeated communications with affected candidates and other stakeholders, and establishment of a taskforce to provide advice and explore the issue. The 53 affected candidates were invited to resit the exam at a later stage free of charge with two additional examination sittings scheduled later in the year ( Australian College of Rural & Remote Medicine, 2020).

Many groups and organizations have considered the implications of the COVID-19 pandemic. The Australian Medical Association Council of Doctors in Training and Specialist Medical College Trainee Chairs and Representatives met in August 2020 to discuss College initiatives underway to allow vocational trainees to succeed with examinations and progress through training during COVID-19 ( Australian Medical Association, 2020).

Attendees acknowledged the significant disruption to examination processes caused by COVID-19 and the impact of prolonged stress on trainees and College staff. While appreciating the efforts made by Colleges, supervisors and examiners to support trainees’ progress through training the group recommended that ‘ensuring the safety of trainees, supervisors, examiners and the public; maintaining the validity of examinations; and providing a consistent examination experience to the extent this is possible, were paramount when taking action to support as many trainees as possible to progress through training.’

The communique from this trainee forum called for:

• A contingency plan involving ‘a robust back up examination delivery option’
• Formalised and ongoing College engagement with the Trainee Committee
• Appropriate stable and tested IT connection
• Robust governance and oversight of exam planning and contingencies

The importance of having an organizational “digital incident response plan” (akin to an emergency response plan/business continuity plan) cannot be overstated. Such a plan needs to consider what committee/group structures are ready to be stood up and how a timely plan can be implemented to troubleshoot, with levels of escalation as needed, i.e. what is the recovery time? There are significant parallels with the pandemic response in healthcare: variations in response to an emerging crisis can lead to significant variations in outcome. Jurisdictions who took early precautionary actions had smaller and less sustained waves of infection. Similarly, terminating a troubled digital assessment early may prevent more significant complications. Establishing an “early warning system” to identify when a digital assessment is at risk allows for early intervention.

Ensuring clear, open and timely communication in the event of a ‘failure of stability’ is equally critical. All organizations hope they will never have to enact such plans, but failure to have a plan is not conscionable in 2021. While there are many stakeholders to consider, the affected trainees and their representatives are paramount. The Trainee Committee can provide invaluable advice, intelligence and advocacy as part of the institution’s response.

The recent trainee forum communique made further recommendations about a number of responses including: trainee support; the maximum allowable time of delay before adopting the backup exam modality; rescheduling exams; refunds and managing costs associated with re-sits; personalized support for each candidate to prioritize trainee wellbeing; provisions for additional training time and examination leave; and independent investigation into the cause of any disruption ( Australian Medical Association, 2020).

These are all important considerations in formulating a plan to manage stability of an assessment program.

## The future

More needs to be done to understand the stability dimension. While there are numbers of case-studies emerging these have been reactive to emerging - often ‘unprecedented’ - events. There is no doubt an emerging research agenda that needs to be addressed. The future is digital hence our focus should be directed towards continuous improvement and seizing opportunities while facing the challenges and mitigating the risks.

As we explore future directions the perspectives of stakeholders are vital - trainees in particular but also training providers and others. This offers a wonderful opportunity for collaboration and sharing of experiences, not just across stakeholders within a discipline, but across disciplinary and state/national boundaries. Many medical colleges and universities experienced similar issues in 2020, leading to considerable resources diverted to “reinventing the wheel.” Sharing experience and corporate knowledge is not just good practice but essential. We have a strong medical education community; perhaps we need a digital inter-agency committee in Medical Education.

We need discussion, shared knowledge and creative solutions to governance and communication challenges and further development of dedicated IT solutions. Approaches that are designed for meetings and conferences may not be fit-for-purpose for assessments.

In addition, we need to consider resources. While travel time and costs might be minimised, these are to some degree balanced by an increase in costs in terms of the IT platform, infrastructure and administration. Examiners appreciate the convenience and flexibility of not having to travel in order to examine, but face-to-face examinations also bring benefits in terms of examiner training, collegiality and networking. In order to be sustainable a new balance needs to be struck. Expectations of candidates, examiners and training organizations have also changed. The effects of border/travel restrictions have impacted the ability to run face-to-face events. We have all grown accustomed to uncertainty - and to doing things in different ways, often without the need to travel.

## Conclusion

The COVID-19 pandemic has been a “black elephant” event which has globally forced us to think differently about the status quo of many things, including assessment. It has necessitated a rapid transition to digital delivery which introduces novel risks, encapsulated in the proposed dimension of “stability.” While online exams minimise travel time and costs for candidates, examiners and staff, these benefits are to some degree balanced by an increase in costs in terms of platform, infrastructure, administration and need for risk mitigation. Therefore, the delivery of assessment in the digital age, requires the ‘utility equation’ to be recalibrated and establishment of a new “sweet spot” for each assessment program.

In *Antifragile,* Nassim Nicholas Taleb describes the concept of antifragility, in which disorder and chaos are substrates for growth and flourishing. For an antifragile educational organisation, this moment in history presents an opportunity for clinical assessment to transition away from the traditional event-based (synchronous) examinations to programmatic models of assessment utilising real-time and longitudinal data collection closer to the source of clinical practice (workplace-based) enabled by the necessary use of technology ( Taleb, 2012).

Transitioning away from traditional models of assessment raises further questions that need robust exploration. However, the challenges and disruptions of the COVID-19 pandemic may prove to be a springboard to innovation rather than a time when we had to do the same things digitally with increased risk. We suggest that the multiple challenges posed by the pandemic and multiple global responses provide an urgent call for action to address the notion of stability in assessment: to understand it, to manage it and to plan, so we can continue to provide stable assessments in an unstable world.

## Take Home Messages

• The move to online exams has introduced novel risks, including the risk of IT failure or cyberattacks compromising the stability of the assessment
• These are not ‘black swans’ or rare and unexpected events; educational institutions must anticipate and mitigate these risks
• ‘Stability’ therefore needs to be considered and managed as another component of the assessment utility equation
• To achieve stability, assessment should be designed to match existing constraints, rather than shoehorning old assessment modalities into new delivery platforms

## Notes On Contributors

**Tarun Sen Gupta** is the Chair of the ACRRM Assessment Committee and Professor of Health Professional Education at the James Cook University College of Medicine and Dentistry, Australia. ORCiD: https://orcid.org/0000-0001-7698-1413

**Eugene Wong** is the ACRRM Core Generalist StAMPS Lead Examiner, a Specialist in Rural Generalist Medicine, and Director of Medical Services at Bundaberg Hospital, Australia.

**Deepak Doshi** is the ACRRM MCQ Lead Examiner, Associate Professor at University of Queensland and Griffith University and Chief Medical Officer at West Moreton Health, Queensland, Australia. ORCiD: https://orcid.org/0000-0002-2263-7470

**Richard Hays** is Professor of Remote Medicine and Health, James Cook University, Australia. ORCiD: https://orcid.org/0000-0002-3875-3134

## Declarations

Richard Hays is the editor of MedEdPublish.
