# Supplementary material for: 'Stability' of Assessment: Extending the Utility Equation
Source: MedEdPublish (2016). 2021 Jun 4;10:155. [Version 1] doi: 10.15694/mep.2021.000155.1 (PMC10939557; doi:10.15694/mep.2021.000155.1)
Supplement: Peer review history

# Reviewer report: Reviewer response for version 1

**DOI:** 10.21956/mep.18979.r27190

**Published:** 2021-10-10

**Authors:** Ken Masters

**Affiliations:** 1 Sultan Qaboos University

This review has been migrated. The reviewer awarded 5 stars out of 5 An excellent and well-timed opinion piece that introduces the concept of platform stability in online assessments. While this has been something of concern when online exams were held on campus, the risks of failure were minimal because the environment was well-controlled, and technical teams were usually available at the point of failure (especially the student’s machine). With distance online exams, the situation changes, and so the stability of the technology must be taken into account, and many schools were unprepared for the problems that occurred.The paper does not offer a common, total solution, because there will not be a solution that suits all institutions, and not even all parts of an institution. The paper does, however, raise all the important issues and demonstrates that this is something that cannot be solved by individual teachers; if institutions are serious about pursuing these types of assessments, then they will have to re-think their approaches to physical IT infrastructure and organisation. I can imagine this paper would form an important discussion paper at any institution that is considering implementing online distance examinations.

Reviewer Expertise:

NA

No decision status is available

# Reviewer report: Reviewer response for version 1

**DOI:** 10.21956/mep.18979.r27189

**Published:** 2021-10-03

**Authors:** Julie Hunt

**Affiliations:** 1 Lincoln Memorial University

This review has been migrated. The reviewer awarded 4 stars out of 5 This article offers a timely opinion that van der Vleuten's model of assessment utility should in fact include assessment stability. This opinion is substantiated by citing the risks posed by IT failure, cybersecurity, etc in the COVID-19 era of remote assessments. I think the article is a worthwhile read for those who are designing and running assessments remotely--and potentially for those who are running them on-site but are using technology to aid their delivery in some manner.I disagree with the authors that security was not mentioned by Norcini et al. in 2018, as they did discuss "secure administration" which includes some of the facets discussed by the authors in this manuscript.I found Figure 1 helpful to introduce the facets of technology that can threaten remote delivery of an assessment, but I would have liked to have seen a more specific application of threats (listed in the orange star) on certain aspects of the exam delivery, rather than lumping them all together and directing them all toward multiple arms of the figure.I also would have preferred that the authors include more examples of successful exams and 'black elephant' exam events, particularly outside of Australia. This would improve the article's relevance to readers outside Australia, and may be helpful in identifying patterns of what works and does not work for remote assessment.Overall, I think the authors have made a good case for considering stability while designing and evaluating the utility of exams.

Reviewer Expertise:

NA

No decision status is available
